# Large litter size increases oxidative stress and adversely affects nest-building behavior and litter characteristics in primiparous sows

**DOI:** 10.3389/fvets.2023.1219572

**Published:** 2023-08-22

**Authors:** Juho Lee, Hyeonwook Shin, Janghee Jo, Geonil Lee, Jinhyeon Yun

**Affiliations:** Department of Animal Science, Chonnam National University, Gwangju, Republic of Korea

**Keywords:** antioxidant capacity, hyperprolific sows, loose housing, nest-building behavior, reactive oxygen species

## Abstract

The study examined 24 primiparous sows (Landrace × Large white) and their offspring, which were grouped based on litter size: NORMAL (*n* = 8, average litter size 11.5 ± 1.2), with litter size between 7 and 14, and LARGE (*n* = 16, average litter size 15.9 ± 1.0), with litter size between 15 and 20. Sows were group-housed during gestation, and housed in an adjustable loose housing system (2.4 × 2.3 m) during farrowing and lactation. All the sows were confined in the farrowing crates (0.6 × 2.3 m) for 7 days after the onset of parturition. Saliva samples of sows were collected on days 35, 21, and 7 before farrowing (D-35, D-21 and D7, respectively), and on days 1, 7, and 28 after farrowing (D1, D7, and D28, respectively) to measure the levels of Trolox equivalent antioxidant capacity (TEAC), hydrogen peroxide (H_2_O_2_), advanced oxidation protein products (AOPP), and tumor necrosis factor-alpha (TNF-α). Colostrum samples were collected for oxytocin and prolactin assays. Nest-building behavior (NB) for 24 h before parturition and farrowing was observed through video analysis. The results showed that LARGE sows had higher levels of H_2_O_2_ on D1 and D7 and AOPP during late gestation (*p* < 0.05, for all) and lower TEAC levels during late gestation and on D7 and D28 after farrowing (*p* < 0.05, for all) than NORMAL sows. Additionally, LARGE sows tended to have higher levels of TNF-α on D1 and D7 (*p* < 0.10, for both). LARGE sows showed shorter duration and lower frequency of NB during 24–12 h before parturition (*p* < 0.05, for both), and tended to have lower prolactin levels (*p* = 0.10). Furthermore, large sows tended to show longer farrowing duration and higher stillbirth rate (*p* = 0.06, *p* = 0.07, respectively). In conclusion, this study confirmed that large litter size may increase oxidative stress in sows during late gestation and lactation. The data also suggested that this could adversely impact prolactin release, leading to reduced NB.

## 1. Introduction

In recent decades, there has been a growing trend to enhance production efficiency in various farm animals, such as sows, rabbits, and sheep, by promoting the production of larger litters (1–3). However, this increase in litter size has resulted in several negative consequences of both welfare and economic concerns (1). Specifically, research has shown that large litter sizes lead to higher rates of farrowing disorders in sows (1). These negative outcomes have been attributed to diverse factors, including longer farrowing duration, greater variability in piglet birth weights, and increased within-litter weight, all of which are related to limited uterine capacity (4, 5). Moreover, large litter sizes can result in increased oxidative stress in sows due to the overproduction of reactive oxygen species (ROS) during late gestation and lactation, which can have negative impacts on both fetal and mammalian cells, leading to poor farrowing and piglet performance (6, 7).

The expression of nest-building behavior (NB) is crucial for successful farrowing and lactation in peripartum sows (1). Yun and Valros (8) have suggested that this innate behavior is closely related to levels of oxytocin and prolactin, which are critical hormones during the farrowing and lactation processes. During prepartum, NB can be triggered by increased prolactin levels (9, 10), which may enhance oxytocin release in peripartum sows (11). However, human studies have suggested that oxidative stress during pregnancy reduces circulating prolactin and oxytocin levels (12, 13). Since large litter size causes an increase in oxidative stress due to the large energy requirements, the expression of NB could be limited in sows with large litters, ultimately leading to poor farrowing and lactation performance.

Given this background, a better understanding of how maternal hormones are affected by oxidative stress status associated with litter size is needed to improve the farrowing process and piglet survival and growth. Therefore, the current study aimed to assess the oxidative stress status of hyperprolific sows based on litter size and investigate its potential relationship with maternal hormones, behavior, farrowing process, and litter characteristics. We hypothesized that oxidative stress status would differ between sows with large litters and those with smaller litters, possibly with increased ROS production. Additionally, we hypothesized that elevated oxidative stress inhibits prolactin and oxytocin release during the peripartum period, which, in turn, decreases the expression of NB before parturition and further reproductive performance.

## 2. Materials and methods

The experimental procedures were approved by the animal experimental ethics committee of Chonnam National University (approval number: 202106-189).

### 2.1. Animals and housing

A total of 24 primiparous sows (Landrace × Large white) were chosen from a pool of 28 sows within a single farrowing batch, after excluding four sows with a litter size less than seven or with farrowing duration exceeding 10 h. The sows were then categorized into two groups based on litter size, with eight sows having a litter size of 7–14 categorized as NORMAL and 16 sows having a litter size of 15–20 categorized as LARGE. The LARGE group was defined as having a litter size greater than the number of functional teats on the sow, which was 14. Within 48 h after birth, piglets were cross-fostered to ensure an equal number of piglets on each sow. The piglets were exclusively taken from the LARGE group and then placed into sows from the NORMAL group. This allowed for the adjustment of litter sizes to 12.6 ± 2.3 piglets per sow. All sows were fed with a commercial feed twice (10:30 and 15:00) daily at 2.0 kg/day before the parturition. After parturition, the amount of feed was gradually increased by 1.0 kg/day up to 10.0 kg/day. The sows were provided with a gestation diet until they were moved to a farrowing and lactating unit. Afterward, they were fed the lactation diet. The gestation diet contained 3.3 Mcal digestible energy (DE)/kg, 12.8% crude protein, 3.7% crude fat, 4.1% crude fiber, 4.6% crude ash, 0.8% calcium, and 0.4% phosphorus. The lactation diet contained 3.5 Mcal digestible energy (DE)/kg, 14.7% crude protein, 3.2% crude fat, 3.0% crude fiber, 5.2% crude ash, 0.9% calcium, and 0.5% phosphorus. Piglets had free access to water from a piglet water cup. Milk replacer was provided *ad libitum* to all piglets starting from day 7 after parturition (D7) using a milk feeder (Mini Transition Feeder; Baker feeders, United Kingdom). Piglets were weaned on day 28 ± 2 of lactation (D28).

**Figure 1:**
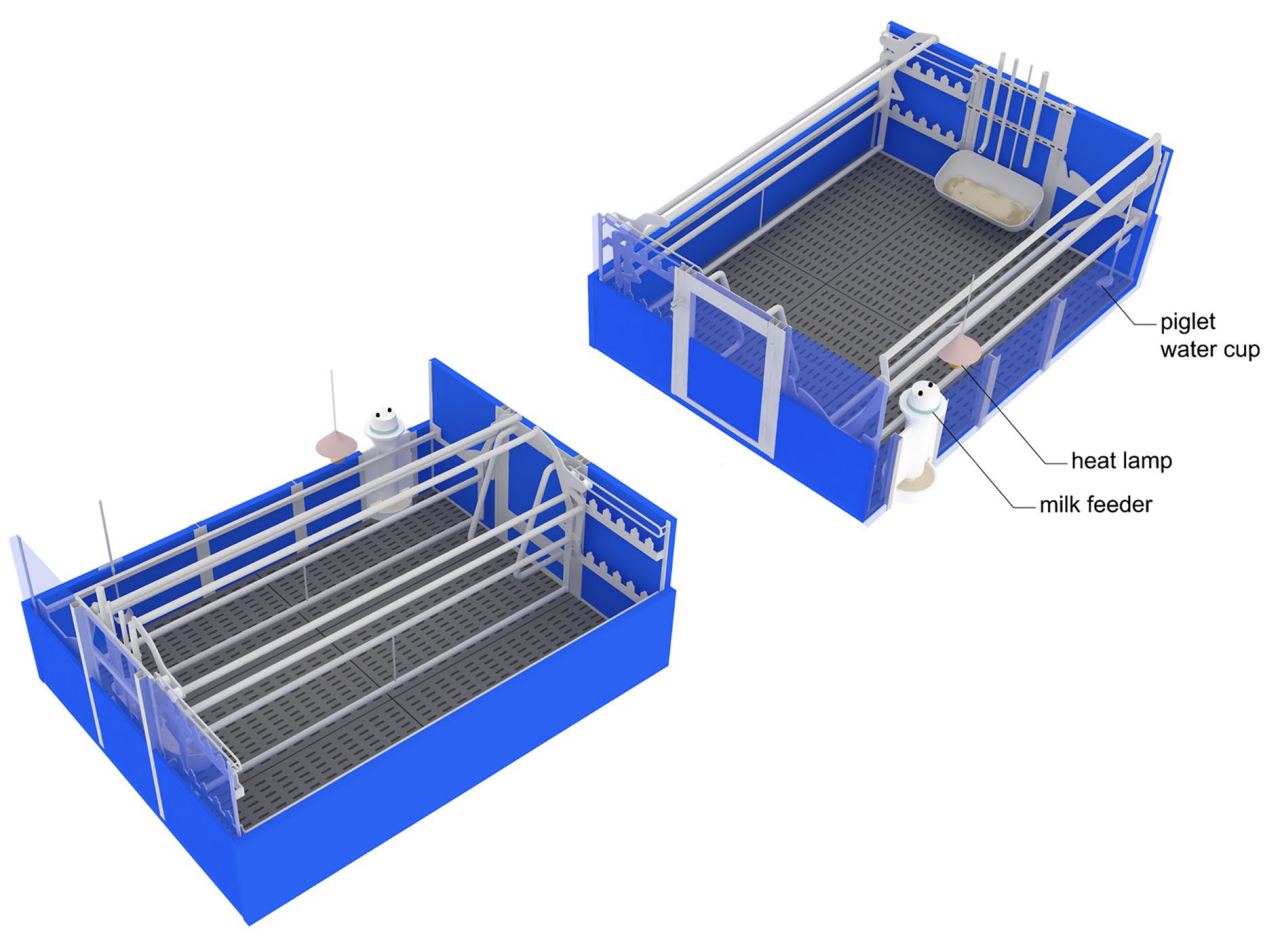
Schematic diagram of the adjustable crate in closed condition (left panel; sow area = 0.6 × 2.3 m, pen size = 2.4 × 2.3 m) and open condition (right panel; sow area = 2.4 × 2.3 m, pen size = 2.4 × 2.3 m).

Before the study, the sows were individually housed in gestation stalls (0.65 × 2.1 m) until day 30 of pregnancy, after which they were moved to a group housing pen (5.75 × 10.5 m) for the remainder of the pregnancy. Approximately 7 days before the expected farrowing date, the sows were moved to a farrowing and lactating room (15 × 15 m) containing 30 farrowing pens. Each farrowing pen (2.4 × 2.3 m) was equipped with an adjustable farrowing crate with a fully slatted concrete floor and heat lamp (Figure 1). The sows were confined to crates (0.6 × 2.3 m) from the onset of parturition until D7 after parturition. During the prepartum and the rest of the lactation period, the sows were housed in loose housing conditions with the farrowing crate opened (2.4 × 2.3 m).

### 2.2. Data collection

#### 2.2.1. Salivary sample collection and assays

Saliva samples were collected from each sow using synthetic swabs (Salivette® Cortisol, Sarstedt, Nümbrecht, Germany) approximately 30 min before morning feeding (10:30 h). The swabs were fixed with curved forceps and placed around the back teeth for approximately 1 min until saturated with saliva on days 35, 21, and 7 before parturition (D-35, D-21 and D-7, respectively) and on day 1 (D1), D7, and D28 after parturition. The collected saliva samples in swabs were stored at −55°C during the sampling period in the pig farm using a deep freezer. The samples were later transferred to the laboratory while maintaining −20°C using a portable freezer and centrifuged for 10 min at 10000 g for subsequent analysis of the Trolox equivalent antioxidant capacity (TEAC). They were also centrifuged for 10 min at 1500 g for analysis of concentrations of hydrogen peroxide (H_2_O_2_), advanced oxidation protein products (AOPP), and tumor necrosis factor-alpha (TNF-α). The supernatant was pipetted into pre-labeled 1.5-mL tubes and stored at −80°C in the deep freezer until the assay. Salivary TEAC and concentrations of salivary H_2_O_2_, AOPP, and TNF-α were measured using ELISA kits (MybioSource, MBS169313; MybioSource, MBS841818; MybioSource, MBS0028634; R&D systems, Duoset #DY690B, respectively). Dilution was not used, and the instructions of the manufacturer were followed. The detection ranges of TEAC, H_2_O_2_, AOPP, and TNF-α were 0–300 μmol/μL, 0–5 nmol/μL, 6.25–200 μmol/L, and 31.3–2000 pg./mL, respectively.

#### 2.2.2. Behavioral observation

All sows and their offspring were video-recorded for a 48-h period, beginning from 24 h before parturition to 24 h after the initiation of parturition, to observe prepartum NB and piglet birth during farrowing. Internet protocol (IP) cameras (HN0-E60; Hanwha Techwin, South Korea) were placed at a height of 1.5 m above the sows’ heads 7 days before the expected farrowing date. The sequence output was recorded by the IP-camera software (Smart Cam Lite v.1.3.2, Hanwha Techwin, United States) and examined with a media player (Naver series on player, Naver corp., South Korea) by two trained researchers. One researcher observed half of NORMAL and LARGE groups, while the other researcher observed the remaining NORMAL and LARGE groups. The inter-observer reliability (IOR) between the researchers was assessed through the Cronbach alpha reliability test, yielding a high value of 0.97, indicating a significantly acceptable level of agreement. The display resolution was 1920 × 1,080 pixels, and the frame rate was 30 FPS. Nest-building behavior was observed for 24 h before the initiation of parturition. The onset of NB was determined when sows performed rooting, arranging, or pawing actions for >5 s, and the end was defined as no performance for >30 s. The researchers involved in NB observations were unaware of which sows were allocated to LARGE or NORMAR groups. The definition of rooting, manipulating and pawing is described below:

- Rooting: pushing the floor using the snout.
- Arranging: manipulating the structure of the pen, including crate and trough, using the snout or mouth.
- Pawing: trying to scrape the floor using the front legs.

The farrowing duration of sow was defined as the time from the birth of the first to the last piglet, including stillbirth. In addition, we calculated birth interval as the time between the births of two consecutive piglets, including stillbirth.

#### 2.2.3. Colostrum sample collection and oxytocin and prolactin assays

Within 3 h of the birth of the first piglet, approximately 30 mL of colostrum was collected from each of the functional teats of all sows using a 50-mL conical tube (SPL, Pocheon, South Korea) for approximately 5 min. A trained experimenter entered the pen cautiously to perform the collection. The collected colostrum samples were stored at −55°C until the end of the experiment. The colostrum samples were then transported to the laboratory while maintaining −20°C constantly with the aid of a portable freezer. Subsequently, the samples were centrifuged at 15,000 rpm for 15 min, and the resulting supernatant was pipetted into pre-labeled 1.5-mL tubes and stored at −80°C in the deep freezer for the further quantification of oxytocin and prolactin. The concentrations of oxytocin and prolactin were determined using ELISA kits (Elabscience, E-EL-0029; MybioSource, MBS2087044, respectively) with a detection range of 15.63–1,000 pg./mL for both oxytocin and prolactin. No dilution was employed, and the procedure for ELISA was followed by manufacturer’s instructions.

#### 2.2.4. Total-born, live-born, stillbirth piglets and piglet birth weight

Since the researchers were present during each parturition, the numbers of total-born piglets, live-born piglets, and stillbirths were separately recorded at birth. Stillbirth was defined as a piglet found dead at birth, with no respiratory action or movement of limbs or body. Mummified piglets were excluded from the experiment. We measured the piglet body weight at birth. They were lifted, dried with dry powder, weighed using an electric scale, and then returned to the pick-up point.

### 2.3. Statistical analysis

The statistical analysis of the data was performed using SAS version 9.4 (SAS Institute Inc., Cary, NC, United States) according to a completely randomized design using sow groups as a fixed effect. The experimental unit was the sow or litter, and data are presented as means ± SEM. To test the normality of the data, PROC UNIVARIATE with the Shapiro–Wilk test was used.

For the analysis of the data on oxidative stress parameters, a PROC GLM model was used. A repeated measure test with a compound symmetry structure model was used for the data analysis for late gestation (D-35, D-21, and D-7), D1, D7 and D28. A PROC GLIMMIX model with a Poisson distribution was fitted to the data for the duration and frequency of NB per 2 h during 24 h before parturition. Random statement with residual option was used in PROC GLIMMIX model. Colostral oxytocin and prolactin levels were analyzed using the PROC GLIMMIX model with a Lognormal distribution. Two colostral prolactin samples from LARGE sows were excluded from analysis due to missing data during the pre-treatment process. A Lognormal distribution was fitted to the PROC GLIMMIX model to analyze the data on farrowing duration, while the data on birth interval were evaluated using the PROC GLM model. The number of total-born piglets was analyzed using the PROC GLIMMIX model with a Poisson distribution, and for stillbirth rate, piglet birth weight, and within-litter birth weight coefficient of variation (CV), the PROC GLM model was fitted. The correlation data were analyzed using Spearman’s rank correlation coefficients (*r*). The oxidative stress data analyzed by repeated measures during the total sampling period were used to determine correlations with prolactin levels, the number of total born piglets and stillbirth rate.

## 3. Results

### 3.1. Salivary oxidative stress parameters

**Figure 2:**
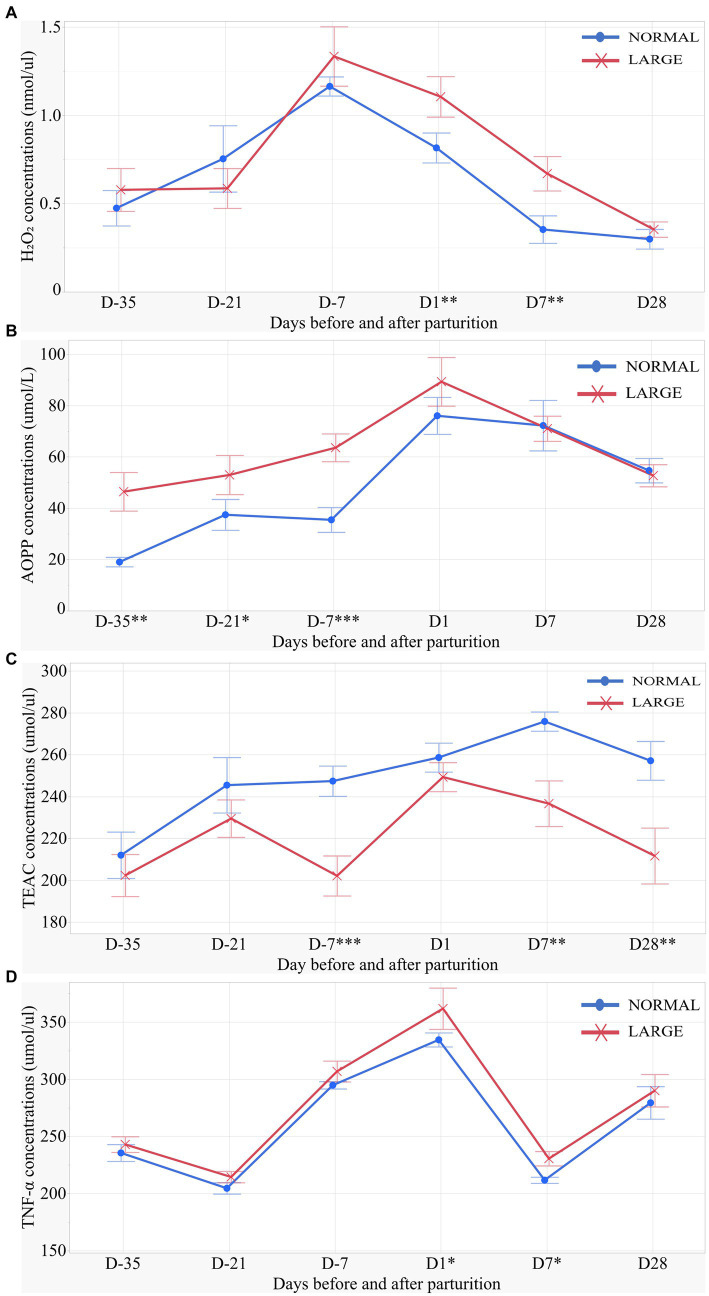
Salivary oxidative stress parameters of sows for **(A)** salivary H_2_O_2_ (hydrogen peroxide), **(B)** salivary AOPP (advanced oxidation protein products), **(C)** salivary TEAC (Trolox equivalent antioxidant capacity), and **(D)** salivary TNF-α (tumor necrosis factor-alpha) levels on days 35, 21 and 7 (D-35, D-21 and D-7, respectively) before parturition and on days 1, 7 and 28 (D1, D7 and D28, respectively) after parturition between normal litters (NORMAL, *n* = 8) and large litters (LARGE, *n* = 16). All salivary samples were collected before feeding time at 10:00 h. Values represent means and SEM. *p < 0.10, **p < 0.05, ***p < 0.01.

LARGE sows exhibited significantly higher H_2_O_2_ levels on D1 and D7 than NORMAL sows (*p* < 0.05 for both, Figure 2A). Repeated measures analysis revealed that AOPP levels were higher in LARGE sows than in NORMAL sows during late gestation (*p* < 0.05, Figure 2B). Additionally, the TEAC levels of LARGE sows were significantly lower than that of NORMAL sows during late gestation as well as on D7 and D28 (*p* < 0.05 for all, Figure 2C). Furthermore, LARGE sows showed a tendency toward higher TNF-α levels on D1 and D7 compared with NORMAL sows (*p* = 0.09, *p* = 0.05, respectively, Figure 2D).

### 3.2. Prepartum NB

**Figure 3:**
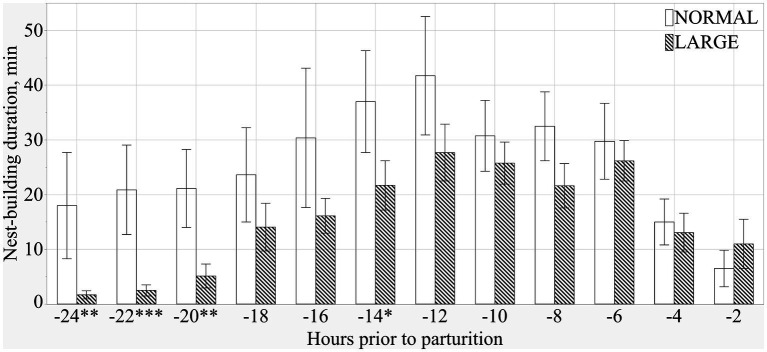
Duration of prepartum nest-building behavior at every 2 h from 24 h before parturition until the birth of the first piglets in normal litters (NORMAL, *n* = 8) and large litters (LARGE, *n* = 16).

Both NORMAL and LARGE sows showed peak NB 12 h before the initiation of parturition (Figure 3). During the 24 h before parturition, LARGE sows showed shorter NB duration than NORMAL sows (means ± SEM; 307.3 ± 45.3 m versus 186.5 ± 24.9 m, *p* < 0.05), and the frequency of NB tended to be lower in LARGE sows than in NORMAL sows (means ± SEM; 51.9 ± 5.3 versus 41.4 ± 3.4, *p* = 0.09). During the 24–12 h before parturition, LARGE sows showed shorter duration and lower frequency of NB than NORMAL sows (means ± SEM; 151.0 ± 35.0 m versus 61.2 ± 15.8 m, *p* < 0.05, and 22.0 ± 3.8 versus 13.3 ± 2.1, *p* < 0.05, respectively).

### 3.3. Colostral oxytocin and prolactin

**Figure 4:**
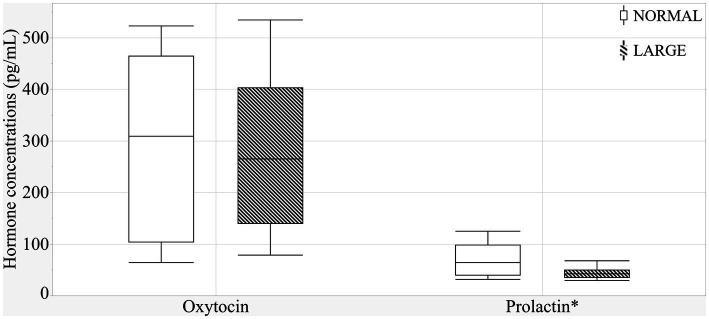
Colostral oxytocin and prolactin levels between normal litters (NORMAL, *n* = 8) and large litters (LARGE, *n* = 16).

The sow groups did not differ in oxytocin levels (Figure 4) (means ± SEM; 293.6 ± 61.7 versus 272.5 ± 35 pg./mL, *p* = 0.97). There was a positive correlation tendency between oxytocin levels and prepartum NB duration (*r* = 0.36, *p* = 0.09). LARGE sows tended to have lower prolactin levels than NORMAL sows (means ± SEM; 69.2 ± 11.5 versus 48.0 ± 5.5 pg./mL, *p* = 0.10, Figure 4), and a significant positive correlation was observed between prolactin and TEAC levels (*r* = 0.25, *p* < 0.01).

### 3.4. Farrowing process and litter characteristics

The farrowing duration of LARGE sows tended to be longer than that of NORMAL sows (*p* = 0.06, Table 1), whereas birth interval did not significantly differ between the two groups (*p* = 0.20, Table 1). The number of total-born piglets was 15.9 ± 1.0 in the LARGE group and 11.5 ± 1.2 in the NORMAL group (Table 1). The stillbirth rate and within-litter birth weight CV tended to be greater in the LARGE group than in the NORMAL group (*p* = 0.07; *p* = 0.06, respectively, Table 1). Piglet birth weight was negatively affected by large litter size, as evidenced by lower birth weight in the LARGE group than in the NORMAL group (*p* < 0.05, Table 1). Farrowing duration tended to be positively correlated with the number of total-born piglets (*r* = 0.40, *p* = 0.05). The number of total-born piglets showed a positive correlation with AOPP levels (*r* = 0.17, *p* < 0.05) and a negative correlation with TEAC levels (*r* = −0.18, *p* < 0.05). Furthermore, the stillbirth rate tended to be negatively correlated with TEAC levels (*r* = −0.13, *p* = 0.09).

**Table 1:** Comparison of farrowing process and litter characteristics between normal litters (NORMAL, *n* = 8) and large litters (LARGE, *n* = 16).

	NORMAL	LARGE	SEM	*p*-value
**Farrowing process (min)**				
Farrowing duration	177.7	264.4	25.0	0.06
Birth interval	19.9	15.5	1.6	0.20
**Litter characteristics**				
Total born	11.5	15.9	0.6	<0.05
Stillbirth (%)	1.0	4.7	1.0	0.07
Piglet birth weight, kg	1.6	1.3	0.1	<0.05
Within-litter birth weight CV, %	17.3	21.0	1.2	0.06

CV, coefficient of variation.

## 4. Discussion

To eliminate potential influences from farrowing experience and environment, we specifically analyzed primiparous sows housed in a single group, as the primary objective of this study was to investigate oxidative stress and maternal response *per se*, while controlling for the potential factors. The present results showed elevated levels of AOPP and H_2_O_2_ in LARGE sows during late gestation and lactation, respectively. These findings indicate increased oxidative stress in sows with large litters. Hyperprolific sows require a greater amount of energy for fetal growth during gestation (14, 15). A previous study on humans demonstrated that energy requirement increases during pregnancy in line with fetal growth (16), which leads to the activation of placental mitochondria that produce superoxide radicals as part of the energy production process (17). Increased energy requirement might be significant in the present study, wherein a positive correlation was found between AOPP levels and litter size. Additionally, earlier studies found that hyperprolific sows experience increased oxidative stress due to increased energy requirements for milk production during lactation (6, 7). Therefore, our current findings suggest that high energy requirements resulting from a large number of fetuses can lead to increased oxidative stress in sows during late gestation, which can continue until weaning.

In the present study, TEAC levels in LARGE sows were consistently lower than that in NORMAL sows during both late gestation and lactation periods. This decrease in antioxidant capacity may be due to an increase in ROS, which leads to an increase in circulating antioxidant utilization to scavenge ROS (18). According to Wang and Walsh (19), the increase in ROS production during pregnancy is a result of activated placental mitochondria producing energy for fetal growth. Myatt and Cui (20) also noted that antioxidant capacity in humans is generally low during pregnancy due to ROS production. Similarly, Berchieri-Ronchi et al. (6) observed reduced antioxidant capacity during late gestation in hyperprolific sows, and our study found a negative correlation between TEAC levels and litter size. Consequently, our findings suggest that high ROS level in LARGE sows results in a decrease in antioxidant capacity during late gestation and lactation periods.

Tumor necrosis factor-alpha is a pro-inflammatory cytokine secreted by innate immune cells and placental cells (21). While normal TNF-α level plays a vital role in regulating progesterone release and supporting implantation, placentation, and embryonic development (21, 22), high TNF-α level can lead to mitochondrial destruction and result in ROS production (19). In our study, we observed a tendency for TNF-α level to be higher during the postpartum period in LARGE sows than in NORMAL sows. Previous studies have reported that limited uterine capacity can cause uterine tissue damage, resulting in an inflammatory response in sows (23, 24). Therefore, our findings suggest that large litter sizes exceeding uterine capacity may cause inflammatory responses during the postpartum period. Additionally, even though TNF-α levels during lactation were lower than during the postpartum period in both sow groups, LARGE sows still showed a tendency for higher TNF-α levels than NORMAL sows during lactation. These results suggest that the recovery of the uterus was delayed in LARGE sows compared with that in NORMAL sows.

Sows typically exhibit peak NB 12–6 h before parturition (10). In the present study, prepartum NB in sows peaked during this time regardless of litter size. Interestingly, however, we observed that LARGE sows showed less NB, particularly 24–12 h before parturition compared with NORMAL sows. This is in contrast to a previous study by Pedersen et al. (25) that reported that sows showed increased prepartum NB as litter size increases. Because the litter size of the sows used by Pedersen et al. (25) was <15, which differs from the litter size of LARGE sows used in our study, a direct comparison of the association with litter size between the two studies may be difficult. NB in sows is initiated by increased circulating prolactin levels before parturition (10). In humans, this level is positively correlated with antioxidant capacity, as prolactin regulates the local production of proopiomelanocortin, which inhibits nitric oxide production (12). Considering that LARGE sows had lower TEAC levels during late gestation than NORMAL sows, we speculate that circulating prolactin levels would also have been lower in LARGE sows, delaying the initiation of prepartum NB and reducing the overall frequency and duration of prepartum NB in them.

The present study indeed observed that colostral prolactin levels tended to be lower in LARGE sows than in NORMAL sows. To the best of our knowledge, there is limited research examining the effects of oxidative stress on prolactin secretion in peripartum sows. In human studies, Vitale et al. (26) reported that the hypothalamic–pituitary–adrenal axis is vulnerable to cellular damage by ROS, ultimately leading to the impaired regulation of hormone secretion, including prolactin releases from posterior pituitary cells (27, 28). Based on these findings, we speculate that excessive production of ROS in LARGE sows caused cellular damage to the hypothalamic–pituitary–adrenal axis, resulting in reduced prolactin secretion. However, further research is needed to confirm the impact of oxidative stress on prolactin secretion in peripartum sows.

During parturition, ROS are generated due to the activation of uterine muscle mitochondria (29). If farrowing is prolonged, this activation is sustained for an extended period, leading to a severe oxidative stress response (30). In the present study, LARGE sows exhibited a tendency for longer farrowing duration compared with NORMAL sows, and there was a tendency for a positive correlation between the number of total-born piglets and farrowing duration. This suggests that prolonged farrowing may result in increased postpartum ROS levels in hyperprolific sows. Although no statistically significant differences in H_2_O_2_ levels during late gestation were observed between the two sow groups, AOPP levels during late gestation, which are indicative of protein oxidation caused by various ROS species including hydroperoxyl radical, superoxide radical, H_2_O_2_, and hydroxyl radical, were higher in LARGE sows than in NORMAL sows. Hydrogen peroxide, which is the third ROS species produced in biochemical processes within the body after hydroperoxyl and superoxide radicals, rapidly reacts with intracellular lipids and proteins to generate hydroxyl radicals (31). Thus, our finding that AOPP levels were elevated in LARGE sows during late gestation suggests that other types of ROS species may also be present at higher levels in this group than in NORMAL sows.

Similar to prior findings by Oliviero et al. (32) and Baxter et al. (4), our study also revealed that LARGE sows exhibited lower piglet birth weight and tended to have higher stillbirth rate and within-litter birth weight CV than NORMAL sows. Uterine blood flow per fetus decreases with increasing litter size, which restricts fetal development by limiting nutrient supply in sows (33). Additionally, Campos et al. (34) reported that excessive large litter size beyond uterine capacity impedes fetal muscle fibre growth, thereby decreasing piglet birth weight and increasing within-litter birth weight variation. Our results therefore support the previous studies suggesting that a large number of fetuses are related to poor fetal development (4). In addition, the stillbirth rate of LARGE sows could be linked to their farrowing duration because prolonged farrowing increases the stillbirth rate by causing hypoxia (4).

We found a tendency that the lower the TEAC levels in sows, the higher the number of stillborn piglets. Oxidative stress in sows impedes fetal development with fetal cell damage, ultimately leading to an increased stillbirth rate (7). A similar human study by Toy et al. (35) has also shown that oxidative stress causes fetal growth retardation, thereby leading to prenatal mortality. Therefore, our finding indicates that oxidative stress in sows is associated with fetal development, which supports a previous study that suggests that oxidative stress decreases reproductive performance in sows (36).

Cross-fostering is a commonly employed practice aimed at effectively managing larger litters, enhancing piglet survival rates, and promoting their overall welfare (37, 38). Studies have suggested that hyperprolific sows experience heightened stress levels during lactation, primarily due to intensified teat competition and heat-related stress arising from the elevated body temperature of piglets (4, 39). This heightened stress can trigger oxidative stress through an elevation in ROS-inducing proinflammatory cytokines (19, 40, 41). In the present study, therefore, the number of piglets on each sow was uniformed in order to mitigate these stressful factors linked to larger litters during lactation. We believe that this cross-fostering procedure helped eliminate variables and ensure a more focused assessment of oxidative stress during the lactation period. Nevertheless, it is important to acknowledge the possibility that sows in the NORMAL group, in particular, could experience additional stress from the introduced fostered piglets.

## 5. Conclusion

The findings of this study provide valuable insights into the impact of increased litter size on oxidative stress levels during later gestation and lactation and prepartum nest-building behavior in sows. These outcomes align with the existing understanding that oxidative stress contributes to the suboptimal farrowing performance observed in hyperprolific sows. Therefore, mitigating oxidative stress emerges as a vital strategy to address the challenges related to large litter sizes in modern pig farms. However, further research will be needed to demonstrate causal relationships between oxidative stress, maternal behavior, and hormonal responses in hyperprolific sows. Future investigations should aim to elucidate the specific mechanisms underlying the impact of oxidative stress on sow performance and explore potential interventions to alleviate oxidative stress and improve the overall well-being and productivity of hyperprolific sows. By expanding our knowledge in this area, researchers and industry professionals can develop targeted strategies to mitigate the negative consequences associated with large litter sizes, ultimately enhancing the welfare and productivity of sows in commercial pig production systems.

## Data availability statement

The raw data supporting the conclusions of this article will be made available by the authors, without undue reservation.

## Ethics statement

The animal study was approved by the animal experimental ethics committee of Chonnam National University. The study was conducted in accordance with the local legislation and institutional requirements.

## Author contributions

JL: project management, practical laboratory, writing and editing, and statistical analysis. HS: laboratory technical help. JJ: project management, practical laboratory. GL: writing and editing. JY: project idea, project funding, funding and project management, intellectual input, writing and editing. All authors contributed to the article and approved the submitted version.

## Funding

This work was supported by the National Research Foundation (NRF) grant funded by the Korea government (2021R1A4A1031220).

## Conflict of interest

The authors declare that the research was conducted in the absence of any commercial or financial relationships that could be construed as a potential conflict of interest.

## Publisher’s note

All claims expressed in this article are solely those of the authors and do not necessarily represent those of their affiliated organizations, or those of the publisher, the editors and the reviewers. Any product that may be evaluated in this article, or claim that may be made by its manufacturer, is not guaranteed or endorsed by the publisher.
